# Exploring the relationship between website quality and equity in living donor kidney transplant

**DOI:** 10.3389/frtra.2024.1490876

**Published:** 2024-11-25

**Authors:** Lisa M. McElroy, Joy E. Obayemi, Brian I. Shaw, Christine Park, Keenan Caddell, LaShara A. Davis, Nicole DePasquale, Dinushika Mohottige, L. Ebony Boulware

**Affiliations:** ^1^ Division of Abdominal Transplant, Department of Surgery, Duke University, Durham, NC, United States; ^2^ Northwestern University Transplant Outcomes Research Collaborative (NUTORC), Comprehensive Transplant Center (CTC), Northwestern University Feinberg School of Medicine, Chicago, IL, United States; ^3^ Department of Surgery, University of Michigan, Ann Arbor, MI, United States; ^4^ Edward Via College of Osteopathic Medicine, Virginia Campus, Blacksburg, VA, United States; ^5^ JC Walter Jr Transplant Center, Houston Methodist Medical Center, Houston, TX, United States; ^6^ Division of General Internal Medicine, Department of Medicine, Duke University, Durham, NC, United States; ^7^ Division of Nephrology, Department of Internal Medicine, Duke University, Durham, NC, United States; ^8^ Department of Medicine, Wake Forest University, Winston-Salem, NC, United States

**Keywords:** living donor kidney transplant, health equity, website, access, readability

## Abstract

**Background:**

Health system websites are important resources to guide health care decisions and may be useful tools to improve racial equity in access to living donor kidney transplant (LDKT).

**Methods:**

We performed a cross-sectional study of adult LDKT programs in the United States. We created an assessment tool for website quality across three domains: accessibility (access to LDKT specific information from the transplant center website), readability (ease of reading and clarity), and educational content (appropriateness and presentation of information, LDKT-specific content, program-specific characteristics, and adherence to equity-centered principles of web design).

**Results:**

Among the 185 transplant center websites reviewed, only 14.6% of LDKT sites could be accessed directly from the transplant center webpage. The median suitability assessment of materials (SAM)—a validated measure of website content for chronic kidney disease (CKD)—was 45 out of 86 (IQR 4) and the median Flesch-Kincaid grade level and ease score were 9.1 (IQR 0.8) on a scale of 0–18 and 51.2 (IQR 5) on a scale of 0–100, respectively.

**Conclusion:**

These results indicate that LDKT websites are currently not available, accessible, and understandable for many potential transplant candidates and donors. Optimizing the content and design of transplant center websites may be a promising and effective strategy for improving equity in access to LDKT.

## Introduction

Today, more than 90% of Americans are regular users of the Internet (1). As such, large numbers of patients look for information about health and healthcare services online (2). Patients use the internet to access information about their illness or disease, learn about medical and surgical treatments, and make decisions about treatment options. Web quality has been demonstrated to influence patient care, engagement, and decision making, positioning health system websites as an important source of health information for patients (3). Internet accessibility is high across a broad range of socioeconomic statuses, and web-based educational content may be an opportunity to improve equity in access to care for patients from marginalized groups or those who require targeted counseling (4). This impels health systems to maximize their efforts to include clear and accurate information on their patient facing websites.

Living donor kidney transplant (LDKT) is the preferred treatment for patients with advanced chronic kidney disease (CKD) (5). Despite a higher incidence of advanced CKD, Black patients are less likely to undergo LDKT and less likely to be kidney donors (6, 7). Racial disparities in LDKT have persisted for decades, and stem in part from limited patient knowledge of LDKT. Studies have identified that having safety information regarding living kidney donation may be an important facilitator for increasing living donation (8). This may be particularly true for donors from racially and ethnically minoritized groups given data demonstrating possible differences in risk profiles for these donors. Websites are therefore an important place where centers can provide population-specific information on LDKT that can empower marginalized communities to have informed conversations with providers and engage in nuanced decision-making around transplantation.

Initial transplant candidate education, donor education, and initiation of the donor evaluation process may all be facilitated via a transplant center's website. However, the extent to which transplant centers use their websites to disseminate this information is unknown. The purpose of this study was to assess the quality of transplant center website information about LDKT. To this end we examined websites of US transplant programs for accessibility, readability, and educational content (i.e., appropriateness and presentation of information, LDKT-specific content, program-specific characteristics, and adherence to equity-centered principles of web design) (9).

## Methods

### Study design and center selection

This was a cross-sectional study of transplant center public facing websites. We included transplant centers in the United States with active accredited adult LDKT programs identified through the Organ Procurement and Transplantation Network (OPTN). Veterans Affairs and children's hospital websites were excluded.

### Website quality metrics and data capture

We created an assessment tool informed by the literature for website quality across three domains: accessibility, readability, and educational content.

We assessed accessibility based on (1) the presence or absence of a link to the transplant center website from the Scientific Registry of Transplant Recipients (SRTR) website, (2) accessibility on a mobile device (categorized as superior, adequate, and inadequate), (3) the presence of a LDKT-specific webpage, (4) the number of clicks required to reach a LDKT webpage from the transplant center home webpage, and (5) the number of LDKT-specific webpages.

We assessed readability using the Readable® website (https://readable.com/) that assesses the ease of reading and clarity of words on a variety of platforms on which contents are presented. The four components of readability were (1) the overall Readable® score (ranging from 1 to 4, with 4 being the highest score equivalent to a Readable® grade of A), (2) the Flesch-Kincaid grade level (equivalent to United States grade level of education with 0 reflecting the level of someone who is just learning to read and 18 representing someone capable of reading an academic paper), and (3) reading ease score (ranging from 0 as extremely difficult to read to 100 as extremely easy to read) were assessed. The fourth component of readability was website compatibility with a mobile device (1 = poor, 3 = excellent, max score of 3).

We assessed website educational content in four main areas: appropriateness and presentation of information, LDKT-specific content, program-specific characteristics, and adherence to equity-centered principles of web design. Appropriateness and presentation of information was evaluated using the suitability assessment of materials (SAM). SAM was previously adapted for chronic kidney disease (10) and examines the message content (11 items, max score 33), text appearance/typography (5 items, max score 15), visuals/graphics (10 items, max score 26), and layout/design (4 items, max score 12) for a maximum total score of 86 [see Table 1].

**Table 1:** Components of LDKT website quality metrics.

Accessibility
(1) SRTR website links to transplant-specific page (2) Accessibility on mobile device (3) Presence of LDKT specific website (4) Clicks to reach LDKT webpage from homepage (5) LDKT-specific web page URL
Readability
(1) Overall readable score (max 4) (2) Flesch-Kincaid grade level (max 18) (3) Flesch reading ease score (max 100) (4) Mobile accessibility (max 3)
Educational content
(1) Appropriateness of information: SAM (max 86) (2) LDKT-specific content (max 17) (3) LDKT program characteristics (max 5) (4) Adherence to equity-centered principles (max 11)

LDKT-specific content was evaluated based on content about treatment options for CKD and ESKD (5 items, max score 5), risks and benefits of LDKT (2 items, max score 2), the kidney donation process (7 items, max score 7), and misinformation clarification (3 items, max score 3) for a maximum total score of 17. These content areas were identified in the literature as being essential information for both donors and recipients when discussing LDKT (11, 12). LDKT program characteristics were evaluated based on whether or not website content identified the LDKT program as existing within the transplant center and the availability of contact information for the program (4 items, max score 4).

Adherence to equity-centered principles was evaluated based on content related to disparities in LDKT treatment based on race or other social determinant of health, information about costs of care or financial support resources, identification of known barriers to LDKT, and national or local community resources (9 items, max score 9) with a maximum total score of 12 [see Table 1]. The choice of these domains was supported by the growing body of literature documenting the extent and nature of inequities in LDKT (13–15).

A codebook was created that encompassed areas of assessment and a team of four trained research staff reviewed the codebook as a group to clarify any ambiguities. Two sets of 20 websites were coded by all team members with discrepancies in coding resolved by consensus. After a final consensus was reached, up to four coders manually reviewed each website (with 20% of all websites coded by all coders) as accessed via the center-specific link provided by OPTN. Each coder explored all available data on the center's website including looking for other associated webpages other than the center-specific link. Readable scores were generated by entering all LDKT related webpages for a given center into the Readable scoring platform. All center scores for each domain were entered into a Research Electronic Data Capture (REDCap) database (16, 17).

### Statistical analysis

We described categorical variables with frequency counts and percentages. We described continuous variables with medians, first quartile, third quartile, and minimum/maximum. An association of SAM with region was examined by ANOVA. All analysis was done using R Studio (Boston, MA).

## Results

### Center characteristics

We reviewed a total of 185 transplant center websites. Based on the 2019 data, the centers had a median deceased donor kidney transplant volume of 67 (Q1 = 38, Q3 = 109, min = 6, max = 329), LDKT volume of 16 (Q1 = 7.5, Q3 = 28.5, min = 1, max = 138), and LDKT black-to-white patient ratio of 0.3 (Q1 = 0, Q3 = 0.4, min = 0, max = 2.9). 46.5% (86/185) of the centers participated in the National Kidney Foundation voucher program (15, 18) and 41.1% (76/185) participated in the paired exchange program (Table 2).

**Table 2:** Center and website characteristics.

Variables	Values (*n* = 185)
DDKT volume, median (Q1,Q3)	67 (38, 109)
LDKT volume, median (Q1,Q3)	16 (7.5, 28.5)
LDKT Black: White ratio, median (Q1,Q3)	0.3 (0, 0.4)
Participation in NKF voucher, *n* (%)	
Y	86 (46.5)
N	99 (53.5)
Participation in paired exchange, *n* (%)	
Y	43 (23.3)
N	142 (76.8)
Accessibility	
SRTR website links to transplant-specific page, *n* (%)	
Yes	43 (23.2)
No	142 (76.8)
Number of clicks to reach LDKT webpage from starting page, *n* (%)	
0–1	27 (14.6)
2	53 (28.6)
3	69 (37.3)
4	28 (15.1)
5 or more	8.0 (4.3)
Number of LDKT-specific web page URL, *n* (%)	
1–4	128 (69.2)
5–7	33 (17.8)
8–10	24 (13.0)
Mobile accessibility, *n* (%)	
Superior	1.0 (0.5)
Adequate	183 (98.9)
Inadequate	0 (0)
Readability	
Overall readable score (max 4), median (Q1,Q3)	3.0 (3.0, 3.0)
Flesch-Kincaid grade level (max 18), median (Q1,Q3)	9.1 (8.3, 10.0)
Flesch reading ease score (max 100), median (Q1,Q3)	51.2 (46.2, 55.6)
Educational content	
SAM (max 86), median (Q1,Q3)	45 (41, 49)
Education (max 17), median (Q1,Q3)	8.0 (4.0, 11.0)
LDKT program characteristics (max 5), median (Q1,Q3)	3.0 (2.0, 3.0)
Adherence to equity-centered principles (max 12), median (Q1,Q3)	3.0 (1.0, 4.0)

### Overall website quality

#### Accessibility

**Figure 1:**
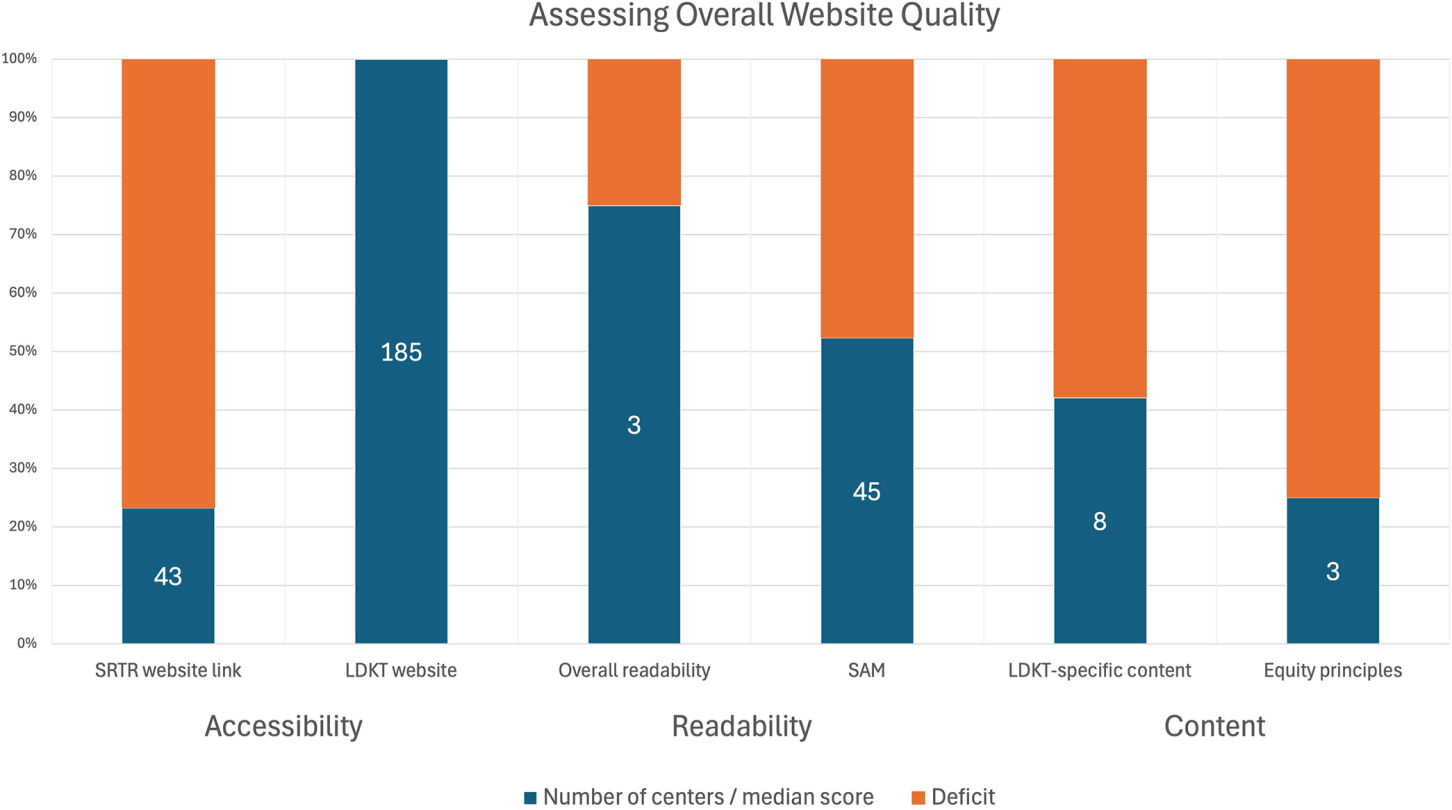
Summary of key outcome measures with number of centers/median score for each measure.

In total, 23.2% (43/185) of the centers had transplant-specific link from SRTR and 98.9% (183/185) demonstrated adequate mobile accessibility. All centers had at least a single page devoted to LDKT, of which 69.2% (128/185) had 1–4 LDKT-specific webpages available on their website. A total of 14.6% (27/185) required 0–1 clicks to reach a LDKT webpage (median 3.0, Q1 = 2.0, Q3 = 3.0, min = 0, max = 5.0). A summary of all website outcome variables and their frequency and/or median score may be found in Figure 1.

#### Readability

The overall median Readable score was 3.0 (Q1 = 3.0, Q3 = 3.0, min = 1, max = 4) and the median Flesch-Kincaid grade level and ease score were 9.1 (Q1 = 8.3, Q3 = 10.0, min = 6.2, max = 12.1) and 51.2 (Q1 = 46.2, Q3 = 55.6, min = 19.8, max = 69), respectively.

#### Educational content

**Figure 2:**
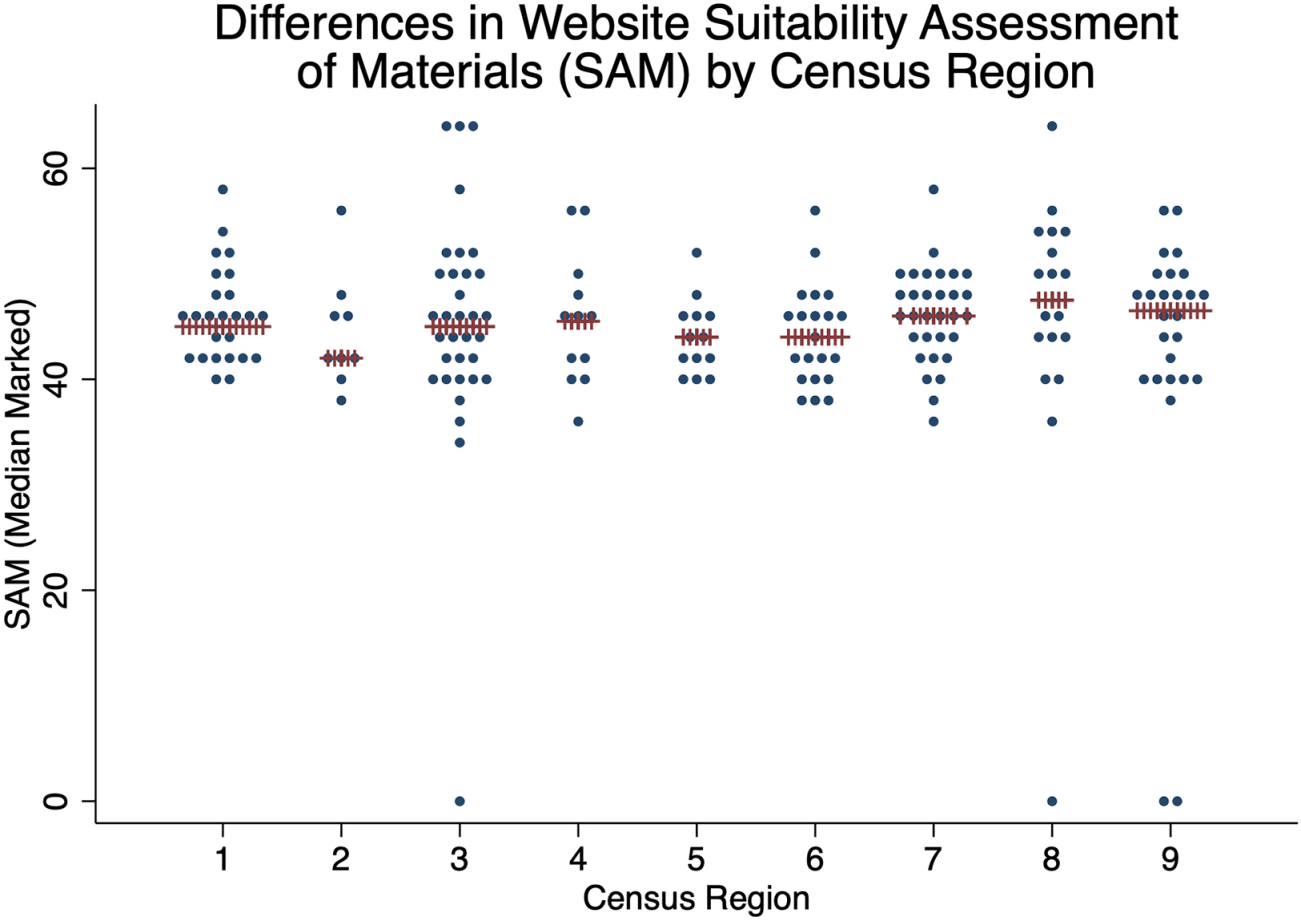
SAM by census region. Median in red X's. There is no association between census region and SAM by ANOVA (*p* = 0.92).

The median SAM was 45 (Q1 = 41, Q3 = 49, min = 34, max = 64), LDKT content score was 8.0 (Q1 = 4.0, Q3 = 11.0, min = 0, max = 14), LDKT program characteristics score was 3.0 (Q1 = 2.0, Q3 = 3.0, min = 0, max = 4.0), and adherence to equity-centered principles was 3.0 (Q1 = 1, Q3 = 4, min = 0, max = 8). We next examined the differences in SAM by Census region and found no difference by region (*p* = 0.92, Figure 2).

## Discussion

We examined 185 websites from active, accredited, adult LDKT programs in the United States and assessed website quality across three domains: accessibility, readability, and educational content. We found nearly universal mobile accessibility, but poor accessibility based on clicks to reach a LDKT webpage. Readability was between the 6th and 9th grade levels with a sign of high reader burden. Most concerning, there was a low level of adherence to principles of health equity within educational content, with almost no information provided about racial disparities in care, financial support, strategies or resources for overcoming individual barriers, or community resources.

Prior work has demonstrated that most patient facing education materials exceed the reading level recommended by the National Institute of Health and American Medical Association (19). In transplant specifically, our study adds to the mounting evidence that points to a need for improvement in patient-facing educational materials about organ transplant. This includes providing educational content in languages other than English (20, 21). A recent review of kidney transplant websites showed that only three websites (1.3%) in 2013 and seven (3.7%) in 2018 reported any evidence of a culturally targeted initiative. In 2018, 35% of centers employed a Hispanic transplant physician, 77% had a transplant physician who spoke a language other than English, and 39% had a transplant physician who spoke Spanish (22). This work was recently highlighted as a primary barrier to equitable care, with a call for more research into how transplant organizations can use web content to reach diverse communities in their referral regions (23).

Recently, increased attention has been devoted to equity-centered culturally responsive health communications. In 2021, Edmons et al. proposed a conceptual framework integrating concepts from positive psychology, critical consciousness theory, and innovation design to support public health programs in integrating culture and social justice into communication and intervention programs (24). The CDC recently released Guiding Principles to Promote an Equity-Centered Approach to Public Health Communication for use by health practitioners developing scientific and other communications (e.g., health education, social media) (9). The guiding principles include recommendations to 1- recognize and reflect the diversity of target audiences, 2-use inclusive and non-stigmatizing language and images, 3-and intentionally consider overlapping individual and system-level contexts. Incorporation of these recommendations into transplant center website content development and design can facilitate more inclusive outreach and health promotion for patients. Though increasing the number of Black donors is key to achieving equity in transplant access, studies documenting that Black donors are less likely to recover pre-donation eGFR and more likely to develop kidney disease after donation may give potential donors pause and are worthy of targeted discussion (25, 26). Patients can be equipped with the knowledge needed to have this discussion through culturally sensitive and equity-conscious information displayed on LDKT websites. Therefore, the use of inclusive content may provide a direct, actionable route to promoting LDKT for patients from a variety of marginalized groups.

Improvement in online educational content about LDKT is an achievable goal and several investigators have developed sites for this purpose (27). One example is Informate’, a bilingual website targeted to the Hispanic community designed to increase knowledge about LDKT among Hispanic patients with end-stage kidney disease, their families, and the public (28). One consideration for the transplant community is linking to external sites such as Informate’ to allow for education without duplicating the time-consuming process of construction. In liver transplant, for example, a number of professional societies have developed websites to provide information on clinical and academic aspects for hepatologists, transplant providers and patients (29).

Racial equity in LDKT is multifactorial but is facilitated by center outreach. Traditional center outreach was in person through health fairs with the public and educational sessions with providers. However, modern center outreach may be digital, and prior work has demonstrated that well-constructed websites and web-based support are effective means of providing transplant education (30) and are well accepted by patients (31–33). Once developed, websites can be leveraged for media-based educational campaigns (34), and bolster caregiver support (35). Web-based self-management for transplant recipients may also help mitigate barriers related to geographic isolation and transportation insecurity (36).

## Conclusion

Transplant center websites are an important but under-utilized tool for patient education and community outreach. Related to LDKT, web-based educational content may be an important aspect of multi-component, multi-level interventions to improve equity in access.

## Data Availability

The raw data supporting the conclusions of this article will be made available by the authors, without undue reservation.
